# Endotension: twenty years of a controversial term

**DOI:** 10.1186/s42155-021-00238-2

**Published:** 2021-06-05

**Authors:** Álvaro Torres-Blanco, Manuel Miralles-Hernández

**Affiliations:** 1 Department of Angiology, Endovascular and Vascular Surgery, Hospital Universitario y Politécnico La Fe, Av/ Fernando Abril Martorell 106, 46026 Valencia, Spain; 2 Department of Surgery, University of Valencia, Valencia, Spain

**Keywords:** Endotension, Abdominal aortic aneurysm, Aorta, Endovascular aneurysm repair, Endoleak

## Abstract

Use of the term endotension in the treatment of aortic aneurysm is currently controversial. Initially it was proposed to define the circumstance in which there is an enlargement of the aneurysm sac after endovascular repair without a demonstrable endoleak. The term was established with the aim of transmitting the possibility of causes other than pressure applying stress to the aneurysm wall. Twenty years have passed since the proposal of this terminology was published. The literature is reviewed with the purpose of providing an update on advances in the knowledge of the possible etiological mechanisms. The experimental studies call into question that causes other than pressure determine the increase of the aneurysm. On the basis of this review, the term `Sac Expansion Without Evident Leak´ (SEWEL) is proposed as a more accurate and precise denomination for what is aimed to be defined. Evidence suggests that the more likely mechanisms of persistent pressurization of the aneurysm sac are an unidentified endoleak (likely type I or low-flow Type II) or thrombus occluding wide and short channels that connects with the excluded aneurysm sac (at the attachment sites of the stent-graft or at the branch vessels orifices).

## Introduction

The term endotension was firstly proposed by Gilling-Smith et al. They defined endotension as “persistent or recurrent pressurization of aneurysm sac following endovascular repair”. They also established a classification of endotension: Grade I was related to type I endoleak, Grade II to type II endoleak, whereas Grade III was related to pressure transmission through the graft (Gilling-Smith et al. 1999). White GH and May J described this scheme as confusing and they defined endotension as “persistent or recurrent pressurization of an aneurysm sac after endovascular graft implantation, without evidence of endoleak”. In the same article they proposed the classification of endoleaks currently in force. In their own words, the term endotension “nicely implies something related to but distinct from endoleak” whereas “conveys the possibility of causes other than pressure applying stress to the aneurysm wall” (White and May 2000). It is important to highlight that tension has several mechanical or physical interpretations, and not only that referred to a fluid pressure. It also defines the state of being stretched, pulled or twisted, and that is the reason why it was deemed more appropriate than endopressure. Be that as it may, the definition of endotension remains a controversial issue. At present, the strict usage of the term is reserved for those circumstances in which there is an aneurysm sac enlargement without a demonstrable endoleak on a delayed contrast computed tomography (CT) scan or other modalities.

Most of aneurysms shrink in size or remain unchanged after endovascular repair. It is widely assumed that the cause is a decrease of pressure within the aneurysm sac. Chuter et al. demonstrated that sac pressure decreases immediately after endovascular repair with aortomonoiliac stent-grafts (Chuter et al. 1997). Sánchez et al. observed the same finding in a canine model (Sanchez et al. 1997) and Parodi et al. in an experimental model using PTFE stent-grafts (Parodi et al. 2001).

In contrast, some aneurysms increase in size following endovascular repair. In most of them, endoleaks are identified. However, some cases of aneurysm enlargement (White et al. 1999; Lin et al. 2003) and even rupture (Kougias et al. 2008) were reported, in which an associated endoleak was not detected. Several hypotheses were proposed to explain these cases. First, pressure could be transmitted to the sac through arterial wall thrombus lining the attachment site of the endograft or through thrombus “sealing” a type 1 endoleak. The pressure could be also transmitted through thrombus originated over the orifices of aortic or iliac branches of the aneurysm. Secondly, one of the most accepted theories is the limitation of current imaging techniques to detect some endoleaks, particularly if the flow is low. Third, another theory proposed the pressure transmission through the endograft wall in the case that material porosity is high. It may also arise through small defects in the fabric of the graft or because of endograft pulsality. Fourth, a pressure buildup from fluid accumulation within the sac was suggested. Seroma-like fluid could accumulate gradually because of thrombus fibrinolysis, graft infection, enzymatic activity or genetic modulation. One last theory proposed that aneurysm enlargement may be independent of pressure.

Twenty years after the proposal of these hypotheses, we reviewed the literature with the purpose of providing an update on advances in the knowledge of the possible etiological mechanisms. The review of the international literature was performed using Medline. The key words “endotension” and “endotension endoleak” were initially used. We found 138 citations from 1999 to 2020. Studies were included in the review if they were related to the concept and etiopathogenesis of endotension after EVAR. Eighteen articles were initially selected. The search was then extended to related articles suggested by the databases and supplemented with searches of reference lists of all relevant articles.

## Review of the literature

### Pressure transmission through thrombus

Certain studies support the theory of pressure transmission though a thrombus or a clot. In an experimental study in a canine model, Marty et al. found that in the group of excluded aneurysms without an endoleak, intraaneurysmal pressure ratio showed a decline to a ratio of 0.34 compared to systemic pressure. In the group of aneurysms with an endoleak, pressure ratio stabilized at 0.75 and the aneurysms remained pulsatile, although the pressure pulse was lower (30 mmHg) than that of untreated aneurysms (62 mmHg). After “sealing” of endoleaks by coil embolization (arteriography and computed tomography confirmed the “sealing”), intraaneurysmal pressure ratio did not decrease (0.76) (Marty et al. 1998).

In another ex vivo study, Mehta et al. created endoleak channels of various lengths and diameters using polytetrafluoroethylene (PTFE) grafts. Peak systolic pressure was recorded in the aneurysm sac, distal to each endoleak channel, before and after the channels were filled with human thrombus. In the absence of thrombus the pressure did not change across the channels, regardless of its length or diameter. In contrast, when an endoleak was thrombosed, pressure reduction was directly proportional to the length and inversely proportional to the diameter of its channel. The authors concluded that thrombosis of endoleaks with short and wide channels may not result in substantial pressure reduction within the aneurysm sac and a successful outcome (Mehta et al. 2001).

### Pressure transmission through the endograft

In an in vitro experimental study, Gawenda et al. analyzed the pressure transmission through the endoluminal graft to a latex aneurysm connected to a circulation model containing a pulsatile pump and a silicone tubing system (Gawenda et al. 2003). The authors found transmission of pulsatile pressure to the latex aneurysm through the graft and they hypothesized that the reason for this phenomenon was what they named “diaphragm effect”. Three different types of grafts were used: thin-wall PTFE, thick-wall polyethylene and thin-wall polyethylene. The conclusion was that transmitted pressure increased with augmenting systemic pressure and it depends on the graft material. Thus, transmitted pressure with PTFE grafts was significantly lower to that recorded with polyethylene grafts whereas pulsatile pressure was lower with low compliance grafts. One important limitation of this study was that commercially available stent-grafts, provided with a wire mesh to enhance columnar strength and radial fixation, were not included. In another experimental in vitro study, they compared the obtained pressures in aneurysm models with 6 or 12 layers, resulting in elastic and stiff compliance. They concluded that pressures were influenced by the compliance (Gawenda et al. 2004).

In contrast, in a more recent in vitro study in a latex model, Bosman et al. demonstrated that pressure transmission through the commercially available stent-grafts wall is clinically irrelevant (Bosman et al. 2009). Seven types of endografts were used: a 3-layer latex tube as reference, a knitted thick-wall Dacron tube graft, a woven thin-wall Dacron tube graft, a thick-wall expanded PTFE tube graft, an Excluder endoprosthesis (WL Gore & Associates, Flagstaff, AZ, USA), a Zenith stent-graft (Cook, Bjaeverskov, Denmark) and a AneuRx stent-graft (Medtronic Vascular, Santa Rosa, CA, USA). The latex reference was used to see if a very compliant “graft” would cause large pressure increases. The thick-wall and the thin-wall Dacron tube grafts, as well as the thick-wall expanded PTFE tube graft, were based on Gawenda’s research. Testing was conducted in an in-vitro pulsatile flow model that was previously described and validated. The systolic and diastolic intra-aneurysm pressures were measured, along with the pulse pressure. The mean intra-aneurysm pressure and pulse pressures were compared for each category of graft (stented/stentless) and for each graft. They found that with increasing systemic pressures, there was a small pressure increase in the aneurysm (< 5 mmHg). In addition, there was no significant difference among the various types of endografts in the dynamic or the static experiments, whereas the pulse pressures were almost identical for all the grafts, not correlating with the stiffness. Therefore, no significant difference in the pressure transmission between stented and stentless grafts was found. According to this finding, the influence of graft rigidity on endotension and the acclaimed “diaphragm-effect” seem less plausible. This study seriously called this effect into question.

### Reliability of imaging methods

In a summary of opinions expressed at an international conference and published in 2002, consensus was reached that some endoleaks could not be detected with even optimal CT scanning. Some authors think that endotension is actually a not identified endoleak by conventional imaging (Lin et al. 2003; Meier et al. 2001; Blackwood et al. 2016). Supporting this theory, there is a reported case of an enlarging aneurysm that was diagnosed as endotension and during open surgery a type III endoleak was demonstrated (Yoshitake et al. 2015).

What seems true is that an ideal imaging technique for endoleak diagnosis is still not available. Duplex ultrasound (DUS), magnetic resonance angiography (MRA), conventional angiography and CT rely on a net movement of fluid or contrast within a certain defined period of time. With each method there is a limit of resolution at which point a small endoleak may remain hidden.

CT has traditionally been considered the gold standard and remains the preferred methodology to evaluate patients. Usually, type I and III endoleaks are detected in arterial phase, whereas type II are detected on a delayed phase. Most CT protocols do not perform delayed imaging with > 180 s postcontrast injection (Rozenblit et al. 2003; Iezzi et al. 2006). However, some studies recommend a delayed CT protocol of up to 300 s to identify low flow endoleaks (Iezzi et al. 2008). Recently, some authors have advocated for using a single-acquisition split-bolus protocol, with simultaneous acquisition of arterial and delayed phase imaging, which could reduce radiation dose by up to 43% (Javor et al. 2017). Photon-counting detector (PCD) CT is an emerging technology, with potential application in EVAR surveillance. The acquisition of CT images at greater than two energy bins allows for better tissue discrimination (Dangelmaier et al. 2018). Improved tissue and material discrimination with PCD CT has potential for both better visualization and dose reduction in the evaluation of endoleaks.

MRA is an alternative, but it requires caution if the stent-graft skeletal is made of steel. Furthermore, the endograft material can influence study quality because stainless steel cause significantly more susceptibility artifact that may preclude optimal assessment. To detect an endoleak, one study with 52 patients found an increased sensivity of 92.9% using magnetic resonance compared with 44% sensivity with biphasic CT, calling into question the superiority of CT (Pitton et al. 2005). Moreover, a meta-analysis showed MRA to be potentially more sensitive than CTA for the detection of endoleaks, particularly for type II endoleaks (Habets et al. 2013). Four-dimensional phase contrast MRA has the capacity to visualize flow dynamics within the aorta, and increased sensivity for the detection of endoleaks relative to CTA (Katahashi et al. 2019; Sakata et al. 2016).

Otherwise, DUS does not require nephrotoxic contrast or radiation. Several studies on color duplex ultrasound (CDUS) and contrast-enhanced ultrasound (CEUS) have had conflicting opinions regarding their diagnostic value relative to CTA. In a meta-analysis, CDUS sensivity to detect type I and III endoleaks was 0.83 and specificity was 1 (Karthikesalingam et al. 2012). The use of ultrasound contrast agent may allow identifying endoleaks that are not detected with CT (Napoli et al. 2004). Thus, some authors think that CEUS may replace CT in surveillance programs after EVAR (Bredahl et al. 2016). A meta-analysis of 42 studies found CEUS to be superior to CDUS for ruling in endoleaks (Abraha et al. 2017). Similarly, in another meta-analysis of 18 studies the authors found that CEUS had higher sensivity and comparable specificity to CTA for the detection of endoleaks (Harky et al. 2019). According to this, a systematic review found that CEUS and MRA are more accurate than CT for the detection of endoleaks, but they are not better than CT for detecting types I and III endoleaks specifically (Guo et al. 2016).

Regarding capability of angiography to detect endoleaks, a comparative analysis showed a sensivity of 63% whereas sensivity with CT was 96% (Armerding et al. 2000). More recent studies found a sensivity between 69% (Ashoke et al. 2005) and 86% (Manning et al. 2009). In the setting of an endoleak identified on the previously cited imaging methods, angiography is an essential modality for further diagnostic characterization and treatment.

Interestingly, and regarding the limitations of angiography, Blackwood et al. created an in vitro model in an experimental study. Measurements of pressure and angiography images were recorded in three scenarios: no endoleak, type I endoleak with inflow and sac outflow and a type I endoleak with inflow but no sac outflow. In the second scenario, aneurysm sac pressure was lower than the systemic and the endoleak was visible at 30 s. In the last scenario sac pressure was higher than the systemic so that net flow was zero and visibility of an endoleak was confirmed after 9 min. Consequently, they concluded that the endoleak could only be visualized with markedly delayed imaging and not with standard angiography like that used in clinical practice (Blackwood et al. 2016). Therefore, endotension may represent an undiagnosed endoleak, particularly type I.

### Fabric porosity

The possible influence of fabric porosity in the pressure transmission to the aneurysm sac is another controversial point. Initially it was proposed as one of the possible causes of endotension although afterwards it was considered as type IV endoleak.

Available endografts are made of different materials and each one has its corresponding porosity grade. Initially, some clinical data suggested that PTFE stent-grafts could not prevent the sac enlargement despite of the aneurysm exclusion in the absence of endoleak. Moreover, some studies observed a lower incidence in the regression of the aneurysm sac in patients that underwent treatment with the original Excluder stent-graft in comparison with other devices (Cho et al. 2004; Bertges et al. 2003; Rhee et al. 2000; Trocciola et al. 2006). Because of the publication of these findings, the Excluder endograft was modified in 2004, incorporating an additional low-permeability layer to reduce porosity.

In an experimental study in a canine model, Trocciola et al. found that stent-graft treatment reduces intra-aneurysmal pressure to < 30% of systemic pressure (nonpulsatile). However, significantly greater pressure was observed after exclusion with PTFE stent-grafts compared with Dacron grafts (Trocciola et al. 2006). Histology showed that those aneurysms that were excluded with the original Excluder stent-graft (thin-wall ePTFE) contained poorly organized thrombus and fibrin deposition, which could be indicative of active remodeling and continued influx of transudated serum. In contrast, aneurysms excluded by Dacron stent-grafts resulted in thrombi that were well organized and chronically composed mostly of granulation tissue. Dense mature collagenous connective tissue was also found in this group.

Haider et al. compared the sac behavior after aneurysm treatment with the original Excluder device, with the low-permeability Excluder device or with the Zenith stent-graft. At 1 year, sac regression rate was 25%, 63.9% and 65.3%, respectively. Consequently, they concluded that low-porosity fabric seems to be an important factor in early aneurysm sac shrinkage (Haider et al. 2006). Reinforcing this conclusion, the long-term results with this new Excluder device confirmed sac regression in 63% at 5 years. Interestingly, sac enlargement was observed only in the setting of a current or previous endoleak, with no cases of hygroma formation noted (Hogg et al. 2011).

A previously cited experimental study compared the new Excluder stent-graft to other available devices (Zenith and AneuRx) and demonstrated that there were no significant differences in the transmitted pressure to the sac among the analyzed devices. In addition, the pulse pressure was identical for all of them (Bosman et al. 2009).

In another study, also in a canine model, Hynecek et al. made a comparison among three distinct stent-grafts: the Trivascular Enovus (nonporous PTFE), the original Excluder (porous PTFE) and the Medtronic AneuRx (Dacron) (Hynecek et al. 2007). Within 24 h after exclusion pulse pressure within the sac tapered to less than 20% of systemic pressure for all three stent graft types. However, throughout the postoperative period significantly lower intra-aneurysmal pressures were present in those aneurysms that were not treated with the porous PTFE device. Histologic analysis of the Excluder-treated aneurysms demonstrated poorly organized fibrin deposition suggestive of acute thrombus. Dacron-treated aneurysms demonstrated mature well-organized collagenous connective tissue. Those aneurysms treated with nonporous PTFE showed characteristics of acute and chronic thrombus. Authors did not find hygromas, although the study period did not exceed 30 days.

Regarding the fabric porosity, it should be underscored that although cases of sac enlargement without a detected endoleak were documented in patients treated with the original Excluder device, the endotension-related rupture incidence was very low. In fact, Kong et al. reviewed data from the multicenter phase I and II clinical trials and reported no endotension-related aneurysm rupture (Kong et al. 2005).

### Fluid accumulation

Regarding the fluid accumulation theory, some cases of hygroma have been reported, describing a gelatinous material within the aneurysm sac (Williams 1998; Risberg et al. 2001; Thoo et al. 2004). One study included four patients with aneurysm sac expansion: one patient had undergone open surgery using a PTFE graft, and three cases had undergone treatment with endografts (two PTFE endografts and one Dacron endograft). The aspirated fluid was described as highly viscous and the analysis reported local hyperfibrinolysis in the sac with signs of local coagulation activation. The authors, Risberg et al., proposed the hygroma theory as a pathophysiological mechanism for endotension (Risberg et al. 2001).

Another study included five cases of symptomatic patients with late sac enlargement, all of them had undergone open repair of abdominal aortic aneurysm using PTFE grafts. Four of them underwent laparotomy and a seroma containing firm rubbery gelatinous material was found in all cases (Thoo et al. 2004). This fact led the authors to suppose that the most likely cause of sac enlargement was the fluid flow from aortic lumen to the aneurysm sac through the graft. It is important to consider that the incidence of symptomatic aneurysm enlargement in the patients after open repair with PTFE grafts was low (2.3%). It also has to be highlighted that the PTFE grafts were thin-walled and differed in porosity compared with PTFE used in the manufacture of Excluder endografts.

### Intermittent endoleaks

Seven cases described as intermittent endoleaks and four cases described as posture-dependent endoleaks were reported in an article (May and Harris 2012). The first case was a patient with aneurysm sac enlargement and no demonstrated endoleak. When the patient underwent reintervention by open surgery, they found a jet of blood when the endograft was subjected to positional changes. They also reported two cases in which the endoleak could only be imaged, using duplex, by changing the patient’s position on the examination table. May et al. concluded that patients with this condition could be considered to have endotension and that the ultrasound would be the most suitable diagnosis test in these cases.

## Discussion

Evidence indicates that aneurysmal sac pressure decreases after endovascular repair. It is widely assumed that this is why most of aneurysms shrink or remain unchanged in size. When there is a persistent endoleak, the intrasac pressure remains high, consequently aneurysms often grow. In some cases, aneurysm sac grows without detecting an associated endoleak. Several hypotheses were proposed to explain these cases. After the review of the literature, some theories have been reinforced whereas others have been weakened.

Evidence derived from experimental studies indicates that pressure is transmitted through thrombus in the case of short and wide occluded channels. Thus, pressure could be transmitted to the excluded aneurysm sac if there is a wide area of thrombus lining the attachment sites of the endograft and the distance to the sac is short. Similarly, the pressure could be also transmitted through thrombus originated over the orifices of aortic or iliac branches of the aneurysm.

Another point to highlight is the limitation of current imaging techniques to detect some endoleaks, particularly if the flow is low. A foolproof imaging technique for endoleak diagnosis is still not available. CT remains the preferred method for diagnosis, but a meta-analysis showed MRA to be potentially more sensitive than CTA for the detection of endoleaks, particularly for type II endoleaks. Furthermore, the results from two meta-analysis and a systematic review concluded that CEUS and MRA can be superior to CT for the detection of some endoleaks. In addition, new technologies such as PCD CT and four-dimensional phase contrast MRA, have potential for better visualization and increased sensivity for the detection of endoleaks relative to CTA. Thus, these new technologies could allow decreasing the number of cases in which the endoleak is not identified. On the other hand, it is also interesting to underscore the limitations of angiography to identify a type I endoleak with no sac outflow. The experimental study of Blackwood et al. demonstrated that this type of endoleak could only be visualized with markedly delayed imaging and not with standard angiography.

In contrast, other etiological theories currently seem less plausible. The experimental study of Bosman et al. determined that the pressure transmission through the commercially available stent-grafts wall is clinically irrelevant and the influence of graft rigidity on endotension is unlikely.

Regarding the fabric porosity theory, it seems unlikely that porosity of the currently available devices is the cause of aneurysmal enlargement. Furthermore, in that case, demonstration with imaging methods would be unlikely as well. However, an endoleak originated by stent-graft fabric rupture could be more likely identified, but they are rare. Similarly, it could be considered that reported cases of fluid accumulation were related to fabric porosity of the PTFE grafts or endografts.

Special mention should be made of the intermittent endoleaks. They were described as depending on postural changes and consequently, there is a limitation of imaging methods for its detection. Given the number of reported cases, it could be considered that they are unusual and sporadic.

Summarizing, experimental studies call into question that “causes other than pressure” apply stress to the aneurysm wall conditioning its enlargement. For this reason, “endopressure” would be a better fit to the concept that it is aimed to be defined, but this term would also include those cases with a detected endoleak. On the other hand, sac expansion can be objectified, but sac pressurization is not objectified throughout conventional follow-up after EVAR. This is why the use of another term such as “Sac Expansion Without Evident Leak” (SEWEL) would be more precise than endotension.

## Conclusions

Evidence suggests that the most likely mechanisms of persistent intra-sac high pressure are two: the endoleak occur but it is not identified (probably a type I or low-flow type II) or pressure is transmitted through thrombus in the case of short and wide occluded channels between the arterial lumen and the excluded aneurysm sac (at the attachment sites of the endograft or through side branches orifices).

On the other hand, type IV endoleak related to fabric porosity would be unlikely with current devices. In the event of this issue, detection by means of the available imaging methods would be unlikely as well.

In our opinion, the used terminology is rather confusing. Given the evidence of existing studies, it would need to be updated. Any of the cited mechanisms in the preceding paragraph may be the origin of a SEWEL (Sac Expansion Without Evident Leak). A detailed analysis of each individual case will allow guidance of the investigation towards any of them.

## Data Availability

Not applicable.

## Abbreviations

CT: Computed tomography
PTFE: Polytetrafluoroethylene
DUS: Duplex ultrasound
MRA: Magnetic resonance angiography
